# The Personal and Health Service Impact of Falls in 85 Year Olds: Cross-Sectional Findings from the Newcastle 85+ Cohort Study

**DOI:** 10.1371/journal.pone.0033078

**Published:** 2012-03-09

**Authors:** Joanna Collerton, Andrew Kingston, John Bond, Karen Davies, Martin P. Eccles, Carol Jagger, Thomas B. L. Kirkwood, Julia L. Newton

**Affiliations:** 1 Institute for Ageing and Health, Newcastle University, Newcastle upon Tyne, United Kingdom; 2 Institute of Health and Society, Newcastle University, Newcastle upon Tyne, United Kingdom; 3 Newcastle upon Tyne Hospitals NHS Foundation Trust, Newcastle upon Tyne, United Kingdom; Innsbruck Medical University, Austria

## Abstract

**Introduction:**

Falls are common in older people and increase in prevalence with advancing old age. There is limited knowledge about their impact in those aged 85 years and older, the fastest growing age group of the population. We investigated the prevalence and impact of falls, and the overlap between falls, dizziness and blackouts, in a population-based sample of 85 year olds.

**Methods:**

*Design*: Cross-sectional analysis of baseline data from Newcastle 85+ Cohort Study. *Setting*: Primary care, North-East England. *Participants*: 816 men and women aged 85 years. *Measurements*: Structured interview with research nurse. Cost-consequence analysis of fall-related healthcare costs.

**Results:**

Over 38% (313/816) of participants had fallen at least once in the previous 12 months and of these: 10.6% (33/312) sustained a fracture, 30.1% (94/312) attended an emergency department, and 12.8% (40/312) were admitted to hospital. Only 37.2% (115/309) of fallers had specifically discussed their falls problem with their general practitioner and only 12.7% (39/308) had seen a falls specialist. The average annual healthcare cost per faller was estimated at £202 (inter-quartile range £174–£231) or US$329 ($284–$377). ‘Worry about falling’ was experienced by 42.0% (128/305) of fallers, ‘loss of confidence’ by 40.0% (122/305), and ‘going out less often’ by 25.9% (79/305); each was significantly more common in women, odds ratios (95% confidence interval) for women: men of 2.63 (1.45–4.55), 4.00 (2.27–7.14), and 2.86 (1.54–5.56) respectively. Dizziness and blackouts were reported by 40.0% (318/796) and 6.4% (52/808) of participants respectively. There was marked overlap in the report of falls, dizziness and blackouts.

**Conclusions:**

Falls in 85 year olds are very common, associated with considerable psychological and physical morbidity, and have high impact on healthcare services. Wider use of fall prevention services is needed. Significant expansion in acute and preventative services is required in view of the rapid growth in this age group.

## Introduction

Approximately 30% of people aged 65 years and over living in the community will fall each year [1], [2] and about two thirds of those in institutional care [3]; 50% of those who fall do so repeatedly [2]. Around 10% of falls will result in serious injury, 6% in fracture [2], [4] and 20% require medical attention [1]. Falls impose a substantial financial burden on health and social care services due to costly hospital and long-term care admissions [5]–[7]. In addition, the psychological consequences of falling impact upon functional status and quality of life [8], [9]. Fear of falling is common in community-dwelling older people with prevalence rates of 26–55% [10]. Whilst a certain amount of caution is necessary in later years, self-imposed activity restriction caused by fear of falling often exceeds safety requirements [11]. This unnecessary activity avoidance may lead to accelerated physical decline, loss of independence and social isolation [8]. A number of studies [12], [13] have shown considerable overlap between falls, dizziness and blackouts with reports suggesting that these may arise due to a common underlying aetiology [14]–[16].

Falls occur more commonly with advancing old age. People aged 85 years and over are now the most rapidly expanding age group in the population with current numbers predicted to double over the next 20 years [17]. A handful of falls studies have focused on this age group [18]–[22]; annual fall prevalence rates in excess of 40% have been reported [18], [19] with recurrent falls occurring more commonly than in the younger old [22]. Although the proportion of falls in this age group resulting in either major soft tissue injury or fracture seems comparable to that in younger community samples, fracture rates appear to increase at the expense of major soft tissue injuries [20]. In addition, very old people are less able to get up without assistance after a fall and may lie on the floor for a prolonged period [23] which brings additional risks of pressure sores, dehydration and hypothermia [24]. Rapid and substantial expansion in the population aged 85 and over will therefore result in marked increases in the overall prevalence of falls and fall-related injuries with important implications for health and social care providers. Data on the impact of falls in this age group on health and social care services is vital to facilitate the planning of future service provision.

In summary, in those aged 85 years and over data is lacking on the impact of falls on health and social care services, the psychological impact, and the overlap between falls, dizziness and blackouts. To address these gaps, we report findings from the Newcastle 85+ Study, a large population-based study of health and ageing in the very old [25], [26].

## Methods

### Objectives

The objectives of this study were to investigate the prevalence of falls in a population-based sample of 85 year olds together with the physical and psychological impact of falls, and their impact on healthcare services including a headline estimate of the cost burden.

### Participants

The methods for derivation of the Newcastle 85+ Study cohort have been reported [25]–[27]. The sampling frame comprised all people born in 1921, aged around 85 years when recruitment commenced in 2006, who were permanently registered with a participating general practice in Newcastle upon Tyne or North Tyneside National Health Service (NHS) Primary Care Trusts, in North-East England. All 64 general practices in these trusts were approached to participate in the study and 53 (83%) agreed; participating and non-participating practices were similar across key practice variables [26]. General practitioners (GPs) were asked to review patient lists before mail-out and to exclude only those with end-stage terminal illness and those who might pose a safety risk to a nurse visiting alone. Excepting these exclusions, all those remaining in the sampling frame were invited to participate in the study whether living at home or in an institution and regardless of their state of health.

### Description of procedures

**Figure 1:**
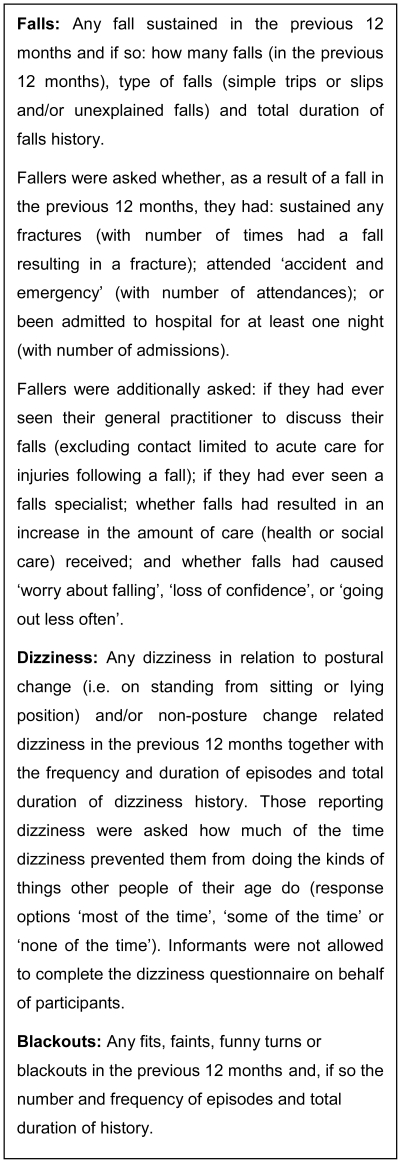
Details of falls, dizziness and blackouts questionnaires.

Participation at baseline entailed a detailed multidimensional health assessment and review of GP medical records; participants could decline elements of the protocol. Assessment was carried out in the participant's usual residence by a research nurse and took place over three separate interviews. Details of the falls, dizziness and blackouts questionnaire (administered in the third interview) are shown in Figure 1; the study questionnaires and the GP record review proforma are available on the study website (www.ncl.ac.uk/iah/research/programmes/85plus.htm). Recruitment and baseline assessment took place over a 17 month period during 2006–7.

### Economic analysis

A simple cost-consequence analysis was performed using unit costs estimated by the Personal Social Services Research Unit, University of Kent, UK [28] to provide estimates of the annual cost burden of falls on health providers (the NHS in the UK). Self-reported data identified those participants who had fallen in the previous 12 months and who reported attending an accident and emergency department (A&E) or staying overnight in hospital as a result of a fall and participants who had ever seen their GP to discuss falls or ever attended outpatients to see a falls specialist. We did not have sufficient information about the reasons for all GP contacts and therefore will not have included all GP fall-related contacts. These services were selected because they account for about 80% of NHS costs for treating older people [30]. Day hospital and community rehabilitation costs which account for some 11% were excluded because of the difficulty of attributing these costs to falls. Costs for each selected service used by participants were calculated by multiplying respective unit costs (at 2007 prices) by the number of service episodes resulting from fall(s). For an A&E attendance the average cost included an estimate of the cost of transporting a person to hospital using an estimate of the cost per fall by the regional ambulance service in 2004 [31] at 2007 prices; the assumption was made that half of the A&E attendances required an emergency ambulance. For an overnight stay in hospital, estimates were calculated by multiplying the number of episodes resulting from fall(s) by the national average (lower - upper quartile) cost of a hospital admission. For those who had fallen in the previous 12 months and who reported ‘ever’ contact with a GP to discuss falls or a falls specialist, an assumption of one GP and/or one falls specialist consultation over the previous 12 months was made. The average costs of these services for those who fell in the previous 12 months were calculated together with the average costs per fall. Where information on use of services was missing for a given participant the appropriate variables were recorded as no use and zero cost for this service. Fall-related costs for the whole sample (fallers and non-fallers) were calculated by setting the costs for non-fallers for each service to zero.

### Ethics statement

The research complied with the requirements of the Declaration of Helsinki. Written informed consent was obtained from participants and where people lacked capacity to consent, for example because of dementia, a formal written ‘consultee opinion’ was sought from a relative or carer according to the requirements of the UK Mental Capacity Act [29]. Details of the consent procedures used in the Newcastle 85+ Study have been reported [27]. Ethical approval was obtained from the Newcastle and North Tyneside 1 Research Ethics Committee (reference number 06/Q0905/2).

### Statistical analysis

Missing values were excluded from the analysis with percentages calculated from the number of valid responses. Denominators vary due to different numbers of missing values. Differences by gender and residence type were assessed using either a Chi-square test (categorical data), ordinal logistic regression (ordinal data) or for continuous non-Gaussian data either a Mann-Whitney U Test (for gender) or a Kruskal-Wallis test (for residence type). Where statistically significant differences were detected, the odds ratio and 95% confidence interval were calculated using logistic regression. Where questions were raised about the reliability of responses (n = 68), for example where there was cognitive impairment and no informant, a sensitivity analysis was carried out with these participants removed; conclusions remained unchanged. All analyses were carried out using Stata 10.1 [StataCorp. 2007. *Stata Statistical Software: Release 10*. College Station, TX: StataCorp LP] with statistical significance at α = 0.05.

## Results

For the Newcastle 85+ Study baseline phase, health assessment data were available on 852 participants (58.6% of the 1453 eligible to participate) [26] and 816 (56.2% of those eligible) completed the falls questionnaire. Due to study drop out, 36 participants in the health assessment did not complete the third interview (when the falls, dizziness and blackouts questionnaire was administered), with remaining loss due to non-completion of specific questions. Data on the overlap between falls, dizziness and blackouts were available for 793 participants.

### Socio-demographics

Of the 816 participants who completed the falls questionnaire, 61.9% (505/816) were female, 77.2% (630/816) lived in standard housing, 12.8% (104/816) in sheltered accommodation (adapted housing with support by home-based services and regular warden visits) and 10.1% (82/816) in a care home. Of those not living in a care home, 60.8% (446/734) lived alone. The recruited sample was broadly representative of 85 year olds in England and Wales in terms of gender, residence in care home and whether living alone [26].

### Prevalence of fallers, number of falls, and duration of fall history

**Figure 2:**
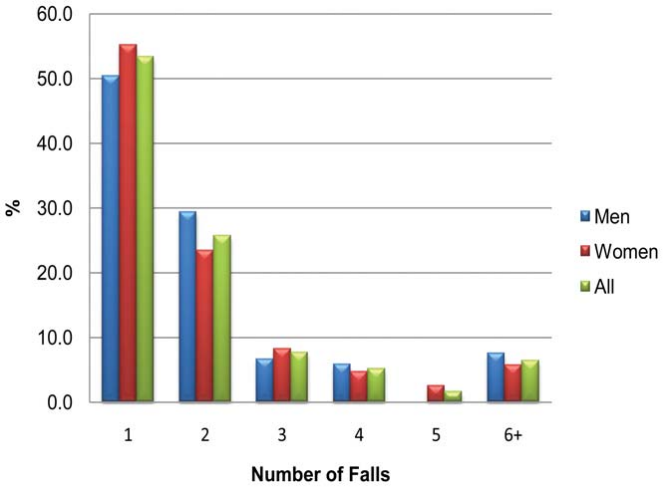
Number of falls reported in the previous 12 months, by gender^1^. Figure footnote: ^1^Reported as percentage of those who have fallen.

At least one fall in the previous 12 months was reported by 38.4% (95% confidence interval (CI) 35.0–42.7%; 313/816) of participants; men 38.6% (120/311), women 38.2% (193/505), Chi-square test of no gender difference p = 0.442. For those who had fallen, the median (inter-quartile range (IQR)) number of falls during the previous 12 months was 1(1–2) (Table 1). Two or more falls were reported by 46.6% (145/311) of fallers, three or more by 20.9% (65/311), and six or more by 6.4% (20/311) (Figure 2). Fallers had been experiencing falls for a median of 12 months, IQR 6–36 months (Table 1). There was no statistically significant difference in the proportion who fell when grouped by place of residence (standard housing 37.8% (238/630), sheltered accommodation 41.4% (43/104), care home 39.0% (32/82); p = 0.780). However, people living in a care home reported a higher number of falls during the previous 12 months than either those in standard housing or those in sheltered accommodation (Table 2).

**Table 1:** Falls: number, duration of history, sub-types, psychological impact and impact on health and social care services - reported for those who had fallen in the previous 12 months, by gender.

	All	Men	Women	p value	Odds ratio (95% CI)*
**Number of falls in previous 12 months, median (inter-quartile range (IQR))**	1 (1–2)	1 (1–2)	1 (1–2)	0.559†	
**Duration of falls history (months), median (IQR)**	12 (6–36)	12 (6–24)	12 (7–36)	0.120†	
**Type of falls in previous 12 months, %(n)** ‡				0.675§	
Simple trip/slip	78.8 (241)	81.4 (96)	77.1 (145)		
Unexplained falls	13.4 (41)	11.0 (13)	14.9 (28)		
Simple trip/slip *and* unexplained falls	4.2 (13)	3.4 (4)	4.8 (9)		
Falls but no slip/trip or unexplained falls	3.6 (11)	4.2 (5)	3.2 (6)		
**Psychological impact of falls, %(n)** ‡					
Worry about falling	42.0 (128)	28.0 (33)	50.8 (95)	<0.001§	2.63 (1.45–4.55)
Loss of confidence	40.0 (122)	21.4 (25)	51.6 (97)	<0.001§	4.00 (2.27–7.14)
Going out less often	25.9 (79)	14.5 (17)	33.0 (62)	<0.001§	2.86 (1.54–5.56)
**Impact of falls on health and social services**					
Fracture due to fall in previous 12 months, % (n)‡	10.6 (33)	8.3 (10)	12.0 (23)	0.308§	
*Number of falls resulting in fracture in previous 12 months, median (IQR)*	1 (1–1)	1 (1–1)	1 (1–1)	0.914†	
Accident and emergency' attendance due to fall in previous 12 months, %(n)‡	30.1 (94)	27.5 (33)	31.8 (61)	0.424§	
*Number of 'accident and emergency' attendances due to fall in previous 12 months, median (IQR)*	1 (1–1)	1 (1–1)	1 (1–1)	0.238†	
Overnight hospital admission due to fall in previous 12 months, % (n)‡	12.8 (40)	13.3 (16)	12.5 (24)	0.830§	
*Number of hospital admissions due to fall in previous 12 months, median (IQR)*	1 (1–1)	1 (1–1)	1 (1–1)	0.211†	
Contact with GP to discuss falls (ever), %(n)‡	37.2 (115)	28.8 (34)	42.4 (81)	0.016§	1.82 (1.09–3.03)
Contact with falls specialist (ever), %(n)‡	12.7 (39)	11.1 (13)	13.6 (26)	0.522§	
Increase in amount of care (health or social) received as result of fall, %(n)‡	9.7 (29)	7.7 (9)	10.9 (20)	0.355§	

* Women: Men, only reported where p value <0.05. CI: confidence interval.

† Mann-Whitney U Test for no gender difference.

‡ Denominator is the number of participants reporting fall in previous 12 months with valid data; denominators vary due to missing values.

§ Chi-square test for no gender difference.

**Table 2:** Falls: number, duration of history, sub-types, psychological impact and impact on health and social care services - reported for those who had fallen in the previous 12 months, by type of residence.

	Standard housing	Sheltered housing	Care home	p value	Odds ratio (95% CI)* sheltered: standard housing	Odds ratio (95% CI)* care home: standard housing
**Number of falls in previous 12 months, median (interquartile range (IQR))**	1 (1–2)	1 (1–2)	2 (1–3.5)	0.029†		
**Duration of falls history (months), median (IQR)**	12 (6–36)	12 (6–36)	24 (12–36)	0.231†		
**Type of falls in previous 12 months, %(n)** ‡				0.915§		
Simple trip/slip	78.7 (185)	76.7 (33)	82.1 (23)			
Unexplained falls	13.6 (32)	16.3 (7)	7.1 (2)			
Simple trip/slip *and* unexplained falls	3.8 (9)	4.7 (2)	7.1 (2)			
Falls but no slip/trip or unexplained falls	3.8 (9)	2.3 (1)	3.6 (1)			
**Psychological impact of falls, %(n)** ‡						
Worry about falling	41.8 (99)	44.2 (19)	40.0 (10)	0.937§		
Loss of confidence	39.5 (94)	44.2 (19)	37.5 (9)	0.818§		
Going out less often	24.8 (59)	38.1 (16)	16.0 (4)	0.096§		
**Impact of falls on health and social services**						
Fracture due to fall in previous 12 months, %(n)‡	9.7 (23)	4.7 (2)	25.0 (8)	0.012§	0.44 (0.10–1.92)	3.04 (1.22–7.57)
*Number of falls resulting in fracture in previous 12months, median (IQR)*	1 (1–1)	1 (1–1)	1.5 (1–2)	0.082†		
Accident and emergency' attendance due to fall in previous 12 months, %(n)‡	29.0 (69)	26.2 (11)	43.8 (14)	0.194§		
*Number of 'accident and emergency' attendances due to fall in previous 12 months, median (IQR)*	1 (1–1)	1 (1–1)	1 (1–2)	0.036†		
Overnight hospital admission due to fall in previous 12 months, %(n)‡	10.9 (26)	11.9 (5)	28.1 (9)	0.023§	1.11 (0.40–3.09)	3.21 (1.34–7.69)
*Number of hospital admissions due to fall in previous 12 months, median (IQR)*	1 (1–1)	1 (1–2.5)	1 (1–1)	0.571†		
Contact with GP to discuss falls (ever), %(n)‡	36.0 (85)	45.2 (19)	35.5 (11)	0.511§		
Contact with falls specialist (ever), %(n)‡	13.1 (31)	7.1 (3)	16.7 (5)	0.440§		
Increase in amount of care received due to fall, %(n)‡	5.6 (13)	14.3 (6)	37.0 (10)	<0.001§	2.71 (0.97–7.62)	9.83 (3.75–25.74)

* Odds ratio calculated only where Chi-square p-value <0.05. CI- confidence interval.

† Kruskal-Wallis test for no difference by housing status.

‡ Denominator is the number of participants reporting fall in previous 12 months with valid data; denominators vary due to missing values.

§ Chi-square test for no difference by housing status.

### Type of falls

Of those who had fallen in the previous 12 months, 78.8% (241/306) reported slip(s) or trip(s), 13.4% (41/306) unexplained fall(s), 4.2% (13/306) both slip(s)/trip(s) and unexplained fall(s), and 3.6% (11/306) neither slip(s)/trip(s) nor unexplained fall(s) (Table 1). The median (IQR) numbers of slips/trips and unexplained falls during the previous 12 months were 1(1–2) and 2(1–3) respectively.

### Psychological impact of falls

Of those who had fallen in the previous 12 months, ‘worry about falling’ was experienced by 42.0% (95% CI 36.4–47.5%; 128/305), ‘loss of confidence’ by 40.0% (95% CI 34.5–45.5%; 122/305), and ‘going out less often’ by 25.9% (95% CI 21.0–30.8%; 79/305) (Table 1). All three psychological impacts were reported more commonly by women compared to men: p<0.001 for each and odds ratios (95% confidence limits) for women to men of 2.63 (1.45–4.55), 4.00 (2.27–7.14) and 2.86 (1.54–5.56) respectively.

### Impact of falls on health and social care services

Of those who had fallen in the previous 12 months, 30.1% (95% CI 25.0–35.2%; 94/312) reported A&E attendance, 12.8% (95% CI 9.1–16.6%; 40/312) hospital admission and 10.6% (95% CI 7.1–14.0%; 33/312) a fracture (Table 1). More than one A&E attendance and more than one hospital admission were reported by 4.5% (14/312) and 1.9% (6/312) of fallers respectively. Compared to those living in standard housing fallers living in a care home were more likely to sustain a fracture due to a fall (odds ratio (95% CI) 3.04 (1.22–7.57); p = 0.017) and to be admitted to hospital (odds ratios (95% CI) 3.21 (1.34–7.69); p = 0.009) (Table 2). Despite the high use of acute healthcare services, only 37.2% (95% CI 31.8–42.6%; 115/309) of fallers had specifically discussed their falls problem with their GP (Table 1). Women were significantly more likely to have discussed their falls problem with their GP, odds ratio (95% confidence limits) for women to men 1.82 (1.09–3.03); p = 0.016. Moreover, only 12.7% (95% CI 8.9–16.4%; 39/308) of those who had fallen in the previous 12 months had ever seen a falls specialist. In 9.7% (95% CI 6.3–13.0%; 29/300) of fallers, having a fall had resulted in an increase in the amount of care (health or social care) received. Fallers living in a care home were more likely to report an increase in the amount of care received compared to those in standard housing (odds ratios (95% CI) 9.83 (3.75–25.74); p<0.001(Table 2).

Table 3 shows health service use due to falls, national average unit costs for those services (with lower and upper quartiles where available), average (lower – upper quartile) 12 month cost per participant (for the sample of fallers and non-fallers combined, N = 816), average (lower – upper quartile) 12 month cost per faller (N = 313), and average (lower – upper quartile) cost per fall (total number of falls = 580). Conversion rates to US dollar and Euro (August 2011) are given as a footnote. The average 12 month cost per faller to the NHS was UK sterling £202 (IQR £174–£231) or in US dollars $329 (IQR $284–$377); when applied to the whole cohort (fallers and non-fallers) this equates to £78 (IQR £67–£89) or $127 (IQR $109–$145) per person. We estimate that the average cost to the NHS of a single fall in this age group is £109 (IQR £94–£125) or $178 (IQR $153–$204); A&E services costs accounted for over half of this (£65, IQR £56–£74 or $106, IQR $91–$121).

**Table 3:** Health service use and 12 month cost of falls.

	FALL-RELATED SERVICE USE		FALL-RELATED COSTS			
Service	%(n) of ‘all’ participants (fallers and non-fallers) in receipt of service due to fall (N = 816)	%(n) of fallers in receipt of service due to fall (N = 313)	National average unit cost (lower-upper quartile)* (£†)	Average cost per participant (fallers and non-fallers) (N = 816) (£†)	Average cost per faller (N = 313) (£†)	Average cost per fall (F = 580) (£†)
Accident and Emergency§	12 (94)	30 (94)	280‡ (241–319)	51 (44–58)	132 (114–151)	65 (56–74)
Hospital admission§	5 (40)	13 (40)	205 (174–246)	12 (10–15)	32 (27–39)	23 (20–28)
Falls specialist outpatient¶	5 (39)	12 (39)	154 (114–190)	7 (5–9)	19 (14–24)	10 (7–12)
General practitioner consultation¶	14 (115)	37 (115)	50#	7#	18#	11#
**Total**	**100 (816)**	**100 (313)**		**78 (67–89)**	**202 (174–231)**	**109 (94–125)**

* Sources: Curtis LA (2007) Unit Costs of Health and Social Care 2007. Personal Social Services Research Unit, University of Kent, Canterbury, UK and Newton JL, Kyle P, Liversidge P, Robinson G, Wilton K, et al. (2006) The costs of falls in the community to the North East Ambulance Service. Emerg Med J 23: 479–481.

† Rounded to nearest pound sterling. Conversion rates (Aug 2011): £1 = US$1.63, £1 = €1.13.

‡ Includes the estimated cost of emergency ambulance use estimated from the average cost of a fall to the regional ambulance service in 2004 (Newton et al. 2006) adjusted for inflation (5% per annum). Lower and upper quartile estimated on basis of Accident and Emergency quartiles (0.86–1.14).

§ Service use in previous 12 months due to fall.

¶ Service use ‘ever’ due to fall.

# Lower and upper quartile not available.

### Dizziness

Dizziness in the previous 12 months related to change in posture (i.e. on standing from a sitting or lying position) was experienced by 31.4% (95% CI 28.2–36.4%; 252/802) of participants and dizziness unrelated to posture change by 17.1% (95% CI 14.5–19.7%; 136/796). A total of 40.0% (95% CI 36.5–43.4%; 318/796) of participants had at least one form of dizziness. Dizziness symptoms had been occurring for a median of 18 months in total, with episodes occurring weekly or more frequently in 52.2% (128/245) of those with postural dizziness and 39.1% (52/133) of those with dizziness unrelated to posture change (Table 4). In the vast majority of participants, individual dizzy episodes lasted a few minutes or less, 89.6% (224/250) for postural dizziness and 81.5% (110/135) for dizziness unrelated to posture change. In over one fifth of sufferers dizziness prevented them from ‘doing the things others of their age do’ for ‘some’ or ‘most of the time’; 21.3% (53/249) for postural dizziness and 25.4% (34/134) for non-posture change related. Women experienced dizziness unrelated to posture change more commonly than men; odds ratio (95% confidence interval) 1.49 (1.00–2.27); p = 0.047.

**Table 4:** Dizziness by type: frequency and duration of history; Fits, faints, funny turns or blackouts: number and duration of history - data reported for those experiencing symptoms in previous 12 months, by gender.

	All	Men	Women	p value
**Dizziness**				
**Frequency of dizziness episodes in previous 12 months, % (n)** *				
*Posture change related dizziness*				0.949†
Daily	25.3 (62)	27.2 (25)	24.2 (37)	
Weekly	26.9 (66)	27.2 (25)	26.8 (41)	
Monthly	20.8 (51)	19.6 (18)	21.6 (33)	
Less than monthly	26.9 (66)	26.1 (24)	27.5 (42)	
*Non-posture change related dizziness episodes*				0.224†
Daily	12.8 (17)	4.8 (2)	16.5 (15)	
Weekly	26.3 (35)	33.3 (14)	23.1 (21)	
Monthly	21.8 (29)	23.8 (10)	20.9 (19)	
Less than monthly	39.1 (52)	38.1 (16)	39.6 (36)	
**Duration of dizziness history (months), median (IQR)**				
*Posture change related dizziness*	18 (7–38)	24 (7–36)	18 (6–48)	0.986‡
*Non-posture change related dizziness*	18 (12–48)	12 (12–36)	24 (6–48)	0.943‡
**Fits, faints, funny turns or blackouts**				
Number of fits, faints, funny turns or blackouts in previous 12 months, median (IQR)	2 (1–6)	2 (1–6)	1.5 (1–5)	0.532§
Duration of fits, faints, funny turns or blackouts history (months), median (IQR)	12 (4–24)	12 (2–24)	12 (4–36)	0.544§

* Denominator is the number of participants reporting dizziness sub-type in previous 12 months with valid data for frequency, denominators vary due to missing values.

† Ordinal logistic regression for gender difference.

‡ Chi-square test for no gender difference.

§ Mann-Whitney U Test for no gender difference.

### Blackouts

Only 6.4% (95% CI 4.7–8.1%; 52/808) of participants had a fit, faint, funny turn or blackout in the previous 12 months; symptoms had been experienced for a median of 12 (IQR 4–24) months in total with a median (IQR) number of episodes over the 12 months of 2 (1–6) (Table 4). In the vast majority (88.2%, 45/51) of those experiencing blackouts, episodes occurred less often than monthly.

### Overlap between falls, dizziness and blackouts

**Figure 3:**
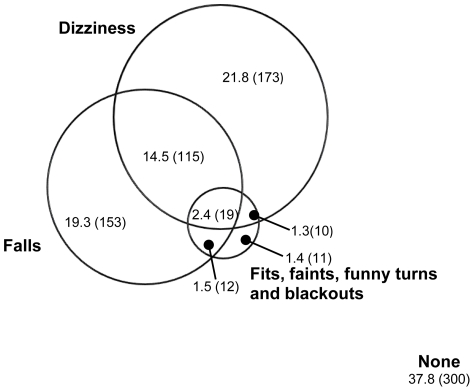
Venn diagram showing the overlap between falls, dizziness and blackouts, % (n)^2^. Figure footnote: ^2^Reported for n = 793 with no missing data in any category.

The Venn diagram (Figure 3, drawn to scale) shows the overlap between participants reporting falls, dizziness, or blackouts. Findings are presented for the sample with valid data on all three symptoms (n = 793) of whom 62.2% (493/793) experienced at least one of the three symptoms. Falls were reported by 37.7% (299/793); around half of these had falls in isolation (51.2%, 153/299) and half in combination with dizziness, blackouts or both (48.8%, 146/299) with the majority of the overlap with dizziness. Dizziness was found in 40.0% (317/793) and again around half had dizziness in isolation (54.6%. 173/317) and half in combination with falls, blackouts or both (45.4%, 144/317), the majority of the overlap being with falls. Blackouts were very uncommon occurring in only 6.6% (52/793), with the majority (78.8%, 41/52) occurring in combination with falls, dizziness or both.

## Discussion

This study of a large population-based sample of 85 year olds has shown that falls are very common in this age group and have significant costs for individuals and healthcare systems. Thirty eight per cent of 85 year olds had at least one fall in the previous 12 months and 11% per cent of fallers sustained a fracture in the 12 month period, which equates to 4% of our 85 year olds overall. Previous studies in this age group report prevalence rates for falls over a 12 month period of 43–44% [18], [19] and fracture rates of 7–9% per fall [20], [21]; our finding that 11% of fallers sustained a fracture in 12 months is not directly comparable as we used ‘fallers’ as our denominator rather than number of falls and almost half of our fallers fell more than once during the 12 months. Surprisingly we did not find a statistically significant gender difference in fracture rates due to a fall despite osteoporosis being commoner in women in our sample [26]. Contrary to previous reports [3], we did not find a higher proportion of fallers in people living in care homes. However, compared to those in standard housing, fallers living in care homes reported a higher total number of falls and were more likely to have sustained a fracture, to have been admitted to hospital, and to report an increase in the amount of health and social care received as a result of a fall.

When we considered the use of medical services as a consequence of falls, we found that overall contact with emergency services was high but contact with preventative services was low. The high hospital admission rate for fallers might reflect the severity of injuries sustained and/or the fact that many in this age group live alone or with an elderly spouse [26] and may therefore be less able to cope at home following a fall. Guidelines on the assessment and prevention of falls in older people [32], [33] recommend that health care professionals should be pro-active in asking older people about falls. Fallers and those at risk of falls should be assessed for gait and balance problems, and multi-factorial risk assessment and individualized interventions implemented in those with falls presenting to medical attention, recurrent falls, gait or balance problems, and those at increased risk of falls; the use of specialised falls services is recommended. Whilst the guidelines state that not all those who fall require multi-factorial assessment and intervention, many experts would contend that all fallers, and those at risk of falls, should be referred. Interventions to prevent falls have been shown to have a significant if modest effect on the risk of further falls [1], [32], [34]. Although firm evidence on the cost-effectiveness of falls services is limited, they have been shown to reduce emergency activity and the length of in-patient stays [35] and analysis conducted by the UK National Institute for Health and Clinical Excellence suggested that both multi-factorial and exercise interventions were cost-effective compared to doing nothing [32]. Our study region has a dedicated falls and syncope service yet it appeared that there was under-referral of fallers in this age group. Whilst we did not know the exact proportion of our fallers who met at least one of the guideline referral criteria, at least 62% did in terms of either recurrent falls or falls requiring medical attention (limited to attendance at A&E or hospital admission) which is considerably higher than the 13% of fallers who were referred. Not all falls were reported to health care professionals. Older people, particularly men, were unlikely to have discussed their falls problem with their GP; possible reasons could be a lack of appreciation that falls are important and that they are not a normal part of ageing. The fact that women were more likely than men to have discussed their falls with their GP might reflect the increased psychological impact of falls in women. Those aged 85 and over are known to have high contact rates with health services, for example 94% of Newcastle 85+ Study participants at baseline had contact with their GP over a 12 month period [26], which provides opportunities for healthcare professionals to be more pro-active in asking about falls and their consequences. It appeared that those fallers who did come to medical attention did not always trigger a referral to a falls specialist; 73% of those who had discussed their falls with their GP had not seen a falls specialist. Possible reasons for under-referral to falls specialist services might be lack of awareness by clinicians that effective strategies exist to reduce falls and associated injuries or older people declining an offer of referral.

The cost consequences to health service providers (the NHS in the UK) of people in later life falling are not insignificant. We estimated that the average cost of a fall to the health service for a person aged 85 was £109 ($178) and the average 12 month per person cost of falls in a representative sample of 85 year olds was £78 ($127). Applying this figure to the number of people aged 80 years and over in England and Wales in 2010 (2.6 million [36]) results in an estimated total annual cost of falls to the NHS for people aged 80 and over of £203 million ($331 million) in 2010. With expansion of the 80+ population [36], this will rise to £377 million ($615 million) by 2030 if per person costs remain constant. In calculating current and projected costs for the 80+ age group, we used the prevalence of falls in 85 year olds as a surrogate for the prevalence in the 80+ age group as 85 is the median age of the 80+ age group in 2010 and for population projections to 2030 [36], [37]. We also made the assumption that the relationship between age and falls prevalence is roughly linear. If the relationship is more exponential and/or the proportion of the 80+ age group aged 85+ increases over time then our figures will be an under-estimate.

We found that falls had significant psychological consequences. Those who sustained a fall lost confidence, worried about falling and as a result limited their activity, reporting that they went out less often. Our rate of fear of falling (42%) was higher than the 32% found in the Leiden 85+ study [38]. Fear of falling is associated with activity avoidance, accelerated physical decline, loss of independence, and social isolation [8] and is predictive of future falls [39], [40]. It is potentially modifiable with simple interventions [41]. GP consultations offer an opportunity to ask about fear of falling and to refer for appropriate interventions. Psychological consequences of falls were significantly commoner in women, in line with previous reports [10], underlining the need to target interventions in a gender specific manner in older people.

To the best of our knowledge, this is the first study to report on the extent of the overlap between falls, dizziness and blackouts in this age group. We found marked overlap pointing to shared underlying patho-physiology or risk factors and underlining the importance of assessing dizziness and blackouts as part of a multi-dimensional review of fall-related risk factors. Dizziness was common in our 85 year olds, particularly postural dizziness, and occurred episodically but frequently and usually lasted only for a few minutes. The presence of this debilitating symptom impacts considerably upon independence in older people. Importantly, postural dizziness and the overlap with falls and blackouts would clinically indicate the possibility of benign paroxysmal positional vertigo and/or orthostatic hypotension in at least a proportion of these 85 year olds. These are both easily diagnosed conditions and potentially curable [14], [42], [43]. Dis-entangling the many possible causes of non-postural dizziness in this age group – such as balance/gait disorders (for example due to sensory deficits, Parkinsonism or medication) and vertigo - was beyond the scope of this study.

This study has a number of strengths. The sample size was larger than in previous studies in this age group and included people in institutional care, who are at increased risk of falls [4], [21] but who have been excluded from some previous studies [18], [20], and those with cognitive impairment. The uniform age of the sample avoided the usual confounding effect of longer female survival when interpreting the sex differences in findings. Assessment was performed in the home setting which is important to ensure a representative sample in an age group who may be unable or unwilling to travel for clinic based assessment. This strategy resulted in a sample which was socio-demographically representative of the local population, and of England and Wales [26]. The availability of data on a range of impacts is an additional strength.

However, it is important also to acknowledge some limitations. The low response rate of 56% should be viewed in the context of the high intensity of the overall study assessment protocol and the advanced age of the participants. In addition to the 56% recruited to the multi-dimensional health assessment, almost all of whom also agreed to review of general practice medical records, a further 13% were recruited to review of general practice records alone with the remainder declining to participate. A comparison of these three groups with respect to socio-demographics and, where possible diagnosed disease, showed little difference between the groups. However, it is possible that non-respondents were frailer than those who participated and hence our estimates of the prevalence and impact of falls may under-estimate the scale of the problem. Data collection relied on retrospective recall over a 12 month period which may result in unreliable estimates; this is a recognised issue in falls and syncope research. Obtaining data on use of health services by self-report may not provide as reliable estimates as direct review of service records, however the latter was not feasible due to resource limitations. Relying on retrospective reporting of recalled falls and use of health services is likely to under-estimate of the problem. Our assessment of dizziness was limited to a questionnaire; we did not conduct a clinical examination or a systematic diagnostic procedure for dizziness. In terms of our cost estimates, we acknowledge that these are crude estimates. Estimating the cost consequences of illness or disease is usually crude because of the large number of assumptions that economists need to make to arrive at an estimate of average cost. It is likely that the costs calculated are an underestimate of the actual costs in this age group since we have been unable to include all health and social services due to the difficulty of attributing many other service costs (e.g. care-home costs) specifically to a fall. Although the accuracy of unit costs estimated annually for UK government departments by the Personal Social Services Research Unit (University of Kent, UK) has improved over the years, they are still based on relatively poor cost data. However despite these limitations, analyses of this kind highlight the significant cost impact of falls to health service providers. Given these caveats we have not attempted to compare our cost estimates with other studies.

### Conclusions

We found that falls were very common in 85 year olds and had a significant impact both on those who fell and on health services. Falls assessment and prevention services were under-used and access to these services needs to be widened. Further research is needed to identify the most effective, and cost-effective, elements of falls assessment and prevention programs, together with the optimal model of service delivery, particularly in view of the marked expansion in service provision which will be required to cope with the rapidly increasing ageing population.
